# Pathogenesis of Endometriosis: New Insights into Prospective Therapies

**DOI:** 10.3390/ijms222111700

**Published:** 2021-10-28

**Authors:** Radhika Kapoor, Christina Anna Stratopoulou, Marie-Madeleine Dolmans

**Affiliations:** 1 Pôle de Recherche en Gynécologie, Institut de Recherche Expérimentale et Clinique, Université Catholique de Louvain, 1200 Brussels, Belgium; rk_bt@rediffmail.com (R.K.); christina.stratopoulou@uclouvain.be (C.A.S.); 2 Gynecology Department, Cliniques Universitaires Saint-Luc, 1200 Brussels, Belgium

**Keywords:** endometriosis, pathogenesis, inflammation, estrogen signaling, apoptosis, epithelial–mesenchymal transition, angiogenesis, medical therapy, pharmacological inhibitors

## Abstract

Endometriosis is a female reproductive disorder characterized by growth of uterine cells and tissue in distant sites. Around 2–10% of women experience this condition during reproductive age, 35–50% of whom encounter fertility issues or pain. To date, there are no established methods for its early diagnosis and treatment, other than surgical procedures and scans. It is difficult to identify the disease at its onset, unless symptoms such as infertility and/or pain are present. Determining the mechanisms involved in its pathogenesis is vital, not only to pave the way for early identification, but also for disease management and development of less invasive but successful treatment strategies. Endometriosis is characterized by cell proliferation, propagation, evasion of immunosurveillance, and invasive metastasis. This review reports the underlying mechanisms that are individually or collectively responsible for disease establishment and evolution. Treatment of endometriosis mainly involves hormone therapies, which may be undesirable or have their own repercussions. It is therefore important to devise alternative strategies that are both effective and cause fewer side effects. Use of phytochemicals may be one of them. This review focuses on pharmacological inhibitors that can be therapeutically investigated in terms of their effects on signaling pathways and/or mechanisms involved in the pathogenesis of endometriosis.

## 1. Introduction

Endometriosis is becoming increasingly common but remains a perplexing disease. It is an estrogen-dependent disorder defined by the presence of endometrium-like tissue in any extrauterine site, even those distant to the uterus, including the pelvic peritoneum, ovaries, and bowel and, rarely, extrapelvic locations [1,2,3]. It usually affects women of reproductive age and its prevalence in this population is around 2–10%; accounting for almost 35–50% of women presenting for infertility and pain management [2]. Endometriosis does not show many specific symptoms, which is why it tends to go undiagnosed or is overlooked for a much longer time. It is typically diagnosed and identified at a later stage, when its metastasis or growth outside the uterus usually starts. Endometriosis is generally reported to cause symptoms such as pain, dysmenorrhea, dyspareunia, lower abdominal and/or back pain and, most importantly, infertility, which is a serious consequence of this disease. Indeed, while endometriosis can initially remain asymptomatic for a long period of time, once aggravated, it greatly compromises quality of life in women suffering from this disease. A key reason for delayed diagnosis is a lack of noninvasive methods for its detection. It can be identified during physical examination upon observation of chocolate brown specks by ultrasound and/or magnetic resonance imaging (MRI). Three types of endometriosis have been distinguished based on their location: (1) superficial peritoneal endometriosis, (2) ovarian endometriomas, and (3) deep endometriotic nodules (DENs) [3]. While endometriosis is a benign disease, it acts much like cancer cells in terms of invasion and metastasis.

Identifying the exact pathogenesis of endometriosis has proven both challenging and contentious for gynecology and reproduction specialists, endocrinologists, and researchers, but recent studies have focused on finding answers. Sampson’s retrograde menstruation theory is considered one of the key hypotheses on the pathogenesis of endometriosis [4]. It describes a backflow of menstrual blood and cells inside the peritoneal cavity, initiating attachment of uterine cells to distant sites. There is an abundance of articles reporting multifactorial causes for the onset of endometriosis, as the pathogenesis of this disease appears to include a range of features, including ectopic endometrial tissue, altered immunity, unbalanced cell proliferation and apoptosis, aberrant endocrine signaling, and genetic factors [5]. For this reason, a very thorough and comprehensive understanding is needed to detect and investigate the physiological, cytological, and immunological events that take place in the microenvironment of the uterus in order to characterize the origins and evolution of the disease. 

Various pathogenic mechanisms have been suggested to contribute to the onset of endometriosis and numerous investigations have explored the causes behind its development. These include physical factors such as uterine tissue damage or scarring (occurring during or after surgery), remnant cells from menstrual blood, stem cells, the uterine microenvironment (conducive to tumor generation), and biochemical factors such as oxidative stress, inflammation, hormones, tumor promoting-genes and proteins, and angiogenesis [2,5]. In line with the various reports published to date, these factors alone or in combination are thought to be causative agents for the initiation of endometriosis. 

In this review, we will focus on the key mechanisms responsible for inducing endometriosis and examine how their crosstalk affects disease development. We will also explore how these mechanisms and specific molecules can be used to develop novel therapeutic options against endometriosis.

## 2. Inflammatory Molecules

Inflammation is one of the main mechanisms that triggers any disease where cell metastasis and invasion are implicated. The scenario for endometriosis, involving cell proliferation and infiltration, is therefore similar. In the uterine environment, the basic functioning of all immune cells—such as macrophages, natural killer cells, and T cells—is regulated by associated increases in the levels of proinflammatory mediators such as prostaglandins, metalloproteinases, cytokines, and chemokines. Proinflammatory pathways prevent apoptotic pathways from clearing and flushing out debris from the system, so these unwanted cells subsequently adhere to distant sites. Basic pathways that regulate adhesion, proliferation, and angiogenesis create a favorable environment for tumor or lesion generation. These immune molecules not only promote propagation and adhesion but also help in the evasion of immunosurveillance, which is one of the major reasons for adhesion of cells in ectopic sites [6]. Various inflammatory molecules are indeed responsible for both initiation of endometriosis and its associated symptoms, such as pain and infertility. Important inflammatory molecules that play a critical role in endometriosis are described below. 

### 2.1. Macrophages

These immune cells are responsible for the detection of foreign elements in the system and their subsequent destruction. Macrophages act by presenting antigens to T cells, and initiate the process of inflammation by releasing cytokines that activate other cells. Macrophages are pivotal in endometriosis, as they are instrumental in lesion adhesion, proliferation, and vascularization, as well as angiogenesis and the innervation process. They also play a role in the sensitization of nerves, which generates pain in endometriosis patients [7]. Activation of macrophages with increased secretion of cytokines has been reported by many researchers. Studies suggest that the levels of various growth factors in ectopic sites of endometriosis patients are elevated compared with those in the peritoneum or peritoneal fluid of healthy controls. Factors such as fibroblast growth factor, macrophage-derived insulin-like growth factor, platelet-activating factor, hepatocyte growth factor, vascular endothelial growth factor (VEGF), and angiogenesis factor have all been reported to be higher in ectopic sites in women with endometriosis than in healthy subjects [7,8,9]. Macrophages participate in proinflammatory cytokine generation, thereby activating pathways of neuroangiogenesis. They are also known for their wide-ranging functional and phenotypic changes. These changes are governed by stimuli including tissue damage, oxidative stress, and hormones, ultimately leading to the activation of different pathways of neurogenesis, angiogenesis, proliferation, migration, and invasion. Macrophage migration inhibitory factor (MIF) and monocyte chemoattractant protein-1 (MCP-1) are among the most potent factors responsible for endometriosis-related inflammation [10]. These factors efficiently and successfully recruit macrophages into endometriotic lesions, which helps them to grow further and proliferate by the release of proinflammatory cytokines and various other growth factors [10,11]. MIF is one such inflammatory factor that plays a crucial role in the early development of endometriosis. It is believed to enhance levels of antiapoptotic proteins during retrograde menstruation and aid survival of cells by activating macrophages, releasing growth factors, and boosting pathways of migration, invasion, and angiogenesis. Studies have reported higher concentrations of MIF in eutopic and ectopic endometrial tissue, peritoneal fluid, and the peripheral blood of endometriosis patients, while a positive correlation has been observed between its expression and serum estradiol levels [12]. Indeed, the literature reports a key role for MIF in the development of endometriosis, especially in the early stages of this disease.

### 2.2. Dendritic Cells

Dendritic cells are a heterogeneous population of antigen-presenting cells that are highly involved in the initiation and modulation of the immune response; as such, their role in the uterus has been specifically explored. Abnormal dendritic cell numbers and functions are correlated with disturbed cytokine and chemokine profiles, including an increase in factors facilitating endometriosis generation and propagation [13,14,15]. They are also thought to be crucial to the generation of pain during endometriosis.

### 2.3. Natural Killer Cells

Natural killer cells are among the most important players in the innate immune system. These lymphocytes act not only against viruses and other pathogens, but also against cancer cells by engaging cytotoxic T cells. Endometriosis patients generally present with lower levels of natural killer cells. Their reduced expression and activity may account for the decreased efficiency in the clearance of endometriotic cells from the peritoneal cavity seen in patients. Reduced activity of natural killer cell ligands and receptors and the presence of inhibitory cytokines in the peritoneum are clearly responsible for the declining performance of natural killer cells and their subsequent inability to clear endometriotic cells from the system [16].

### 2.4. Modulators: Interleukins, Cytokines, Interferons

An important aspect in the development of endometriosis relates to concentrations of prostaglandin in the uterine system, whose biosynthesis is governed by nuclear factor kappa B (NF-κB). NF-κB is a transcription factor regulating innate immunity. At the cellular level, it controls the transcription of DNA, cytokine production, and cell survival. In addition to its crucial role in inflammation and immunity, it is also markedly involved in cell adhesion, invasion, proliferation, apoptosis, and angiogenesis. This makes NF-κB integral to the pathogenesis of endometriosis, as all of these events are fundamental to the onset and development of the disease. NF-κB is even to blame for the increased proinflammatory cytokine concentrations and growth factors seen in the uterine environment. The various roles of NF-κB in endometriosis pathogenesis are summarized in Table 1. 

All of these processes collectively help proliferative cells to settle in distant sites and develop and propagate endometriosis. For this reason, pharmacological inhibitors targeting NF-κB have been proposed as potential therapeutics against the disease. A list of these inhibitors and their targeted pathways can be found in Table 2.

### 2.5. Proinflammatory Cytokines

Cytokines are known to exhibit pleiotropic, cytostatic, chemoattractant, and angiogenic effects, and many are involved in the development and progression of endometriosis. Interleukins (IL-1, IL-33, and IL-8) have been extensively reported in endometriosis, specifically in endometriotic or uterine cells during angiogenesis, migration, invasion, and tumor generation. A number of reports have found that women with endometriosis demonstrate increased expression and release of various proinflammatory cytokines and growth factors—such as IL-1, IL-6, IL-8, epidermal growth factor, and hepatocyte growth factor—in their ectopic and eutopic endometrium and their peritoneal fluid [17,18,27,28]. This activates one or more biochemical processes (including angiogenesis, invasion, and cell proliferation and/or migration). Elevated levels of IL-10, IL-6, IL-8, COX-2, VEGF, and TNF-α have been observed in the peritoneal fluid of endometriosis subjects [28]. The stroma of affected endometrium is associated with increased adhesion of extracellular matrix proteins, MMPs, and elevated IL-8 levels. A representative list of upregulated cytokines and their role in endometriosis pathogenesis can be found in Table 3.

These inflammatory cytokines also play key roles in the progression of endometriosis by promoting survival, growth, invasion, differentiation, angiogenesis, and immune evasion. Tocilizumab and other pharmacological substances antagonizing the effect of these cytokines may be effective in reducing endometriosis-associated inflammation [35] (Table 4).

## 3. Hormones: Estrogen Receptor Alpha (ERα) and Beta (ERβ)

Normal balanced levels of progesterone and estrogen maintain the regular physiology and function of the human uterus and its endometrium through efficient menstrual cycles. Estrogen governs proliferation of the endometrium, while progesterone inhibits the action of estrogen and helps to initiate the process of decidualization. Any imbalance between progesterone and estrogen leads to impaired uterine function. In endometriosis, when cells or tissue from the endometrium attach to non-native sites, there is an altered efficiency of progesterone and estrogen hormones, generally resulting in resistance to progesterone and an excess of estrogen [50,51]. Progesterone resistance can indeed explain why approximately one third of endometriosis patients do not respond to the use of progestins [50]. The excess of estrogen and inevitable imbalance invites local infiltration of immune cells, thus causing inflammation. Inflammation then triggers various factors and helps newly established cells to grow into lesions by activating different pathways of cell proliferation, angiogenesis, metastasis, and invasion. ERs and progesterone receptors (PRs) are responsible for the proliferation and differentiation of normal endometrium. ERα and ERβ work together; however, ERα is less prevalent in endometriosis than ERβ is [51]. ERβ is involved in the simultaneous targeting of many pathways, with prostaglandin synthesis being one of the most important. Prostaglandins give rise to inflammation and prevent apoptosis [6]. In addition, ERβ activates various tumor-promoting and angiogenic proteins and the epithelial-mesenchymal transition (EMT), which later leads to the progression of endometrial cells into lesions. Estrogen has a profound effect on MMP9/2, which participates in the invasion of cells and is also responsible for activating pathways such as Wnt/β-catenin [51]. A change in the expression of estrogen-metabolizing enzymes is also considered a major factor in the pathogenesis of endometriosis and estrogen abundance. PRs function by limiting estrogen levels by means of its receptor ERα. Low concentrations of ERα in endometriosis have been blamed for progesterone resistance in endometriotic lesions, explaining why progesterone is unable to control estrogen levels in endometriosis. 

In theory, the ideal solution would be to lower estradiol levels, while at the same time maintaining sufficient concentrations to minimize side effects including vasomotor symptoms and the loss of bone mineral density. GnRH antagonists block GnRH receptors and thereby cause the suppression of follicle-stimulating hormone and luteinizing hormone in a dose-dependent manner. Donnez and Dolmans previously reviewed the results of different studies on the outcomes of linzagolix, relugolix, and elagolix treatment of symptomatic endometriosis, concluding that these drugs may constitute a promising new treatment option [52]. 

## 4. Apoptotic, Autophagic, and Tumor-Promoting Genes/Proteins

Every redundant cell is fated to be destroyed by the programmed cell death required for effective maintenance of the tissue system, a mechanism called apoptosis. The uterus and endometrium even have an organized system to eliminate unwanted cells by shedding the endometrium under the influence of hormones. Every menstrual cycle involves extensive cell proliferation and apoptosis, with a decrease in progesterone levels when the endometrium is removed from the system. In normal endometrium, there is an observed increase in apoptotic proteins during the late secretory phase. However, endometriosis patients do not experience a rise in apoptotic proteins, indicating that apoptosis is inhibited in endometriosis, shifting the focus to the survival of endometrial cells that then implant in ectopic sites. There could be many reasons why expression of apoptotic proteins is hampered. These include an increase in cytoprotective genes (heme oxygenase-1, NAD(P)H quinone oxidoreductase-1) activated by the NRF2/Keap1 complex in the presence of oxidative stress caused by excess hormones or inflammatory molecules [53]. Autophagic proteins also play a crucial role in the survival of endometriotic cells and, in particular, endometriosis recurrence. It is important to manage the expression of autophagic proteins to limit survival of endometriotic cells. Once they survive and develop into lesions, tumor-promoting genes are activated by various mechanisms and encourage survival of these cells. 

Apoptotic and autophagic pathways may represent promising targets for the development of novel therapeutic options against endometriosis [54]. Table 5 summarizes the apoptotic and autophagic proteins thought to be involved in endometriosis development.

## 5. Epithelial-Mesenchymal Transition

Endometrial cells have the inherent ability to change their structural and functional state from a polarized epithelial phenotype to a highly motile fibroblastoid or mesenchymal phenotype. This is termed EMT and is an important process wherein cells transform their structure and become invasive, as in embryonic development, chronic inflammation and fibrosis, and cancer progression. EMT also causes endometrial cells to develop metastatic and invasive properties. Various biomarkers have been found to play a crucial role in EMT, which is a common process that enables noninvasive (epithelial-like) cancer cells to invade and metastasize (mesenchymal-like). In this process, E-cadherin and N-cadherin are among the most instrumental players. While E-cadherin works to detach endometrial cells from their native site, N-cadherin encourages propagation, invasion, and metastasis. This represents one of the starting points in pathogenesis, turning benign endometriosis into invasive endometriosis, which are often characterized as stages of the disease (Table 6). Decreased E-cadherin and increased expression of mesenchymal markers (N-cadherin and vimentin) are typically observed in endometriotic lesions, leading to reduced cell adhesion and greater cell migration and invasion [62,63]. E-cadherin gene polymorphisms may well be associated with endometriosis [64]. Two factors are primarily responsible for the EMT process in endometrial cells: the first being the hypoxic environment, and the second being estrogen levels [65,66]. Estrogen stimulates the production of angiogenic proteins such as VEGF and increases transcription factors Snail and Slug, all of which later affect E-cadherin expression and enhance the ability of endometriotic cells to spread and metastasize. Hypoxia-inducing factor, on the other hand, initiates EMT through transcription factors zinc finger E-box binding homeobox 1 (ZEB1), Snail, Slug, and the Twist family, known to be key EMT regulators. The activation of various transcription factors under stress conditions has also been observed, most notably including Notch1, transforming growth factor beta (TGF-β), Hedgehog, phosphatidylinositol-3-kinase, and lysyl oxidase. Logically, because of the involvement of EMT in endometriosis progression, pharmacological inhibitors that target these pathways could be beneficial to endometriosis patients (Table 7). 

## 6. Angiogenesis

The process of forming new blood vessels and ensuring a proper blood supply is termed angiogenesis. For endometrial cells and/or debris that have migrated as a result of retrograde menstruation to a distant site and are hypoxic due to an excess of estrogens, the initiation of angiogenesis is important in order for their survival. A compact innervation process is required and is present in endometriotic lesions [76]. New blood vessels are formed from circulating endothelial progenitor cells produced in bone marrow at high levels in lesions. Various factors—including hypoxia-inducible factor 1-alpha and stromal cell-derived factor 1—are known to facilitate the movement of endothelial progenitor cells from bone marrow to sites of angiogenesis. Proinflammatory cytokines such as IL-6 and IL-8 are found at high concentrations in endometriotic lesions and stimulate angiogenesis by attracting neutrophils to these sites. One of the most crucial factors affecting angiogenesis is VEGF [77]. It encourages: cell proliferation, the supply of essential nutrients to growing cells, their migration, the formation of blood vessels, and initiation of the processes of vascularization and even invasion. High levels of VEGF have been reported in endometriotic lesions in rats and mice and peritoneal fluid and tissue samples from endometriosis patients. As new blood vessels form, various growth factors and the MMP complex come into play, including fibroblast growth factor, MMP2, and MMP9, which are important in invasion and metastasis of endometrial cells. Various reports in the literature describe increased expression of VEGF, MMP2, and MMP9 in endometriotic lesions in rats, mice, and peritoneal fluid from patients. There are a number of factors that are also pertinent to angiogenesis, such as platelet-derived endothelial cell growth factor, endoglin, MIF, interleukins, and protein tyrosine phosphatase. A list of pharmacological factors antagonizing the effects of these factors can be found in Table 8. 

## 7. Conclusions

**Figure 1:**
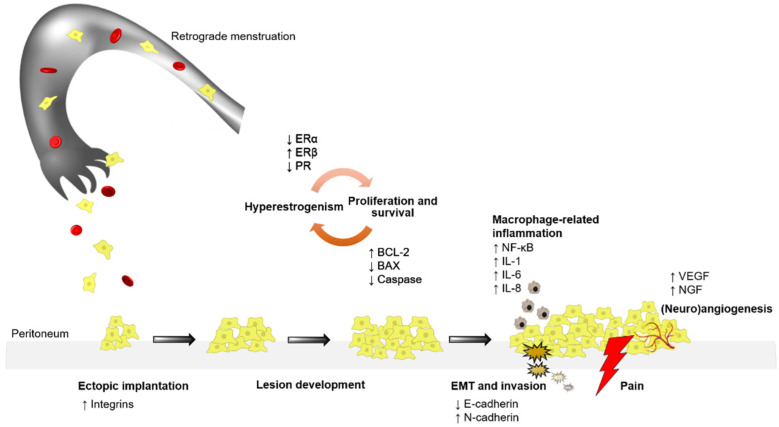
Summary of the mechanisms involved in endometriosis initiation and progression. Endometrial tissue/cells are carried by retrograde menstruation to ectopic locations (peritoneum), where high expression of integrins facilitates their implantation. The constant hyperestrogenism observed in endometriosis, followed by enhanced cell proliferation and survival, leads to lesion development and progression. At the same time, accumulating macrophages release a number of inflammatory mediators, thereby triggering the process of EMT, with the subsequent invasion and angiogenesis/neuroangiogenesis leading to lesion growth and the generation of pain symptoms in endometriosis patients.

Detailed and extensive studies on the subject have led us to understand that the pathogenesis of endometriosis is multifactorial and that each of its factors correlates with the preceding factors (Figure 1). Hyperestrogenism, excessive iron load, loss of apoptotic proteins, enhanced tumor-promoting and invading proteins, and integration of inflammatory molecules are primarily responsible for the pathogenesis of this disease. Factors such as oxidative stress and hormone imbalance create a favorable environment for endometrial cells to metastasize to distant locations and form tumors or lesions, triggering various pathways of inflammation, angiogenesis, and tumor generation. These dysregulated pathways have recently been explored to identify specific therapeutic functions of pharmacological inhibitors that are active in one or more pathways, allowing for the management and abrogation of endometriosis.

## Figures and Tables

**Table 1:** Recent research indicating the impacts of NF-κB on various mechanisms and the subsequent effects on endometriosis development.

NF-κB Role in Different Processes	Reported Key Players in the System	Specific Functions
Effect on angiogenic proteins [17,18,19]	Upregulates angiogenic factors including interleukin 8 (IL-8) and MIF in endometrial and endometriotic cells and VEGF	MIF stimulates endothelial cell proliferation
Effect on invasion proteins [17,18,19]	Matrix metalloproteinases (MMPs), urokinase-type plasminogen activator, and tissue plasminogen activator	These are known to be implicated in remodeling the extracellular matrix, which could lead to endometrial invasion of the submesothelial space of the peritoneum
Effect on cell proliferation [19]	Endometriotic cell proliferation in eutopic and ectopic sites is governed by NF-κΒ, inhibiting apoptosis and favoring the development and maintenance of endometriosis	This primarily activates p50/p65 NF-κB dimers involved in the transcription of genes that regulate innate immunity, inflammation, and cell survival; intercellular adhesion molecule 1, B-cell lymphoma 2 (Bcl-2), and Bcl-XL (antiapoptotic proteins at the mitochondrial level); and caspase 3, caspase 8, and caspase 9
Effect on the inflammatory process [17,18,19]	NF-κB-activated macrophages release proinflammatory cytokines and growth factors involved in boosting inducible nitric oxide synthase, cyclooxygenase-2 (COX-2), IL-1, IL-6, IL-8, tumor necrosis factor alpha (TNF-α), and VEGF	Activation of p65 NF-κB dimers in innate immunity

**Table 3:** Various cytokines and their role in the activation and development of endometriosis.

Name of Cytokine	Specific Functions	Study Model
IL-1/IL-1B [29,30,31,32]	IL-1 is basically responsible for and primarily functions to create a proinflammatory microenvironment in tissue, resulting in fever, inflammation, and even sepsis with the help of various integrins. IL-1 is present in endometriosis generation, where it facilitates the innervation process. Various studies applying different models report altered expression of IL-1 and its relation to disease progression and associated pain. IL-1 is widely linked to infertility caused by endometriosis	Rats Mice ESCs In vitro culture of peritoneal macrophages
IL-2 [32,33,34]	Promotes invasion and migration of ESCs, along with their growth	Women Rats
IL-6 [32,33,35,36]	IL-6 gene polymorphisms have been extensively studied in endometriosis patients, who demonstrate elevated levels of IL-6. Anti-IL-6 monoclonal antibody was found to be restorative against endometriosis in rats	Women Rats
IL-8 [17,37,38]	IL-8 is widely associated with adhesion and propagation of endometrial cells, along with increased expression of proteins involved in migration and invasion. It also promotes the progesterone resistance observed in endometriosis. IL-8 has an inverse relationship with apoptotic genes and proteins, thereby boosting lesion growth	Women Follicular fluid Primary ESC culture Rats
IL-33 [39,40,41]	IL-33 is one of the most crucial players in acute and chronic inflammation. It is known for its active role in all major processes, such as pain development, cell invasion, migration and adhesion, neovascularization, and many others, culminating in endometriosis. Its role is well established in DENs, especially at advanced stages of the disease	Women Rats Mice ESCs
TNF-α [42,43,44,45]	A proinflammatory cytokine known to impair glutathione, resulting in the accumulation of reactive oxygen species. Induces IL-6, IL-8, granulocyte-macrophage colony-stimulating factor, and MCP-1, while enhanced cell proliferation triggers COX-2 expression	Women Rats Mice ESCs

**Table 5:** Important apoptotic and autophagic proteins in the pathogenesis of endometriosis.

Apoptotic, Autophagic and Tumor-Promoting Proteins	Role in the Pathogenesis of Endometriosis/Reason for Interplay	Study Model
Autophagy-related gene 3 [55]	Component of the autophagic mechanism. Estrogen levels and progesterone resistance are also considered to be its major regulators	Mice
Beclin [56,57,58]	Induced by hypoxic conditions in endometrium. Activated in response to progesterone levels	Humans Rats Mice ESCs
Microtubule-associated protein light chain 3 [56,58,59]	Decreased p62 with impaired inactivation of AKT, ERK1/2, and mechanistic target of rapamycin (mTOR)	Humans Mice
Bax/Bcl-2 [55,60,61]	ERβ plays a key role in anti-apoptosis, inflammation and invasion of ectopic lesions, activates mTOR, and demonstrates excessive expression of soluble Fas ligand. Constant source of TNF-α. Suppresses E-cadherin	Humans Mice Endometrial cells
Caspase intrinsic/extrinsic [25,26]	Reduced percentage of apoptotic cells and greater number of surviving cells entering the peritoneal cavity	Mice ESCs

**Table 6:** Role of EMT regulators in the development of endometriosis.

EMT Regulators	Role in Endometriosis	Study Model
E-cadherin [62,63,65,66,67,68,69,70,71]	Allows endometrial cells to detach from their primary site, and also invasive endometrial cells to implant in pelvic sites. Loss of the epithelial cell phenotype, including the basement membrane junction	Humans Baboons Rats Endometrial cells
N-cadherin [62,63,65,66,67,68,69,70,71]	Elevated expression of N-cadherin possibly enhances cell motility by reducing the stability of cell-adhesion complexes	Humans Baboons Rats Endometrial cells
Twist [70]	Specific transcription factor involved in EMT and dedifferentiation, which maintains invasion and metastasis. Increased concentrations found in EMT	Humans
Snail/Slug [65,66,67,68,69,70]	Snail/Slug are known to be associated with loss of differentiation, tumor progression, and metastasis	Humans Rats Endometrial cells
ZEB [72]	ZEB1 is a downstream effector of the TGF-β signaling pathway, which inhibits E-cadherin expression for progression of epithelial tumors. Most commonly seen in DENs. Expressed in lesions but not endometrium	Humans
β-catenin [62,71]	β-catenin was detected in nuclei of epithelial cells in ovarian endometriosis, suggesting activation of the Wnt/β-catenin signaling pathway, a well-known EMT regulator during organ development. Inhibits E-cadherin expression	Humans Endometrial cells

**Table 8:** Effect of pharmacological inhibitors on endometriosis development.

Name of Inhibitor	Mainly Targeted Downstream/Upstream Proteins	Mode of Action	Study Model
Multidrug resistance protein 4 [78]	Wnt/β-catenin	Involved in embryo receptivity by stabilizing endometrial β-catenin	Endometrial cells Mice
Genistein [79]	MMP9, MMP2	Reduces lesion size by targeting MMP signaling	Mice
Fasudil [80]	Rho/Rho-associated kinases	Attenuates myofibroblast differentiation and contractility, decreasing fibrosis. Regulates cell proliferation and apoptosis	EESCs
Sunitinib, SU6668, SU5416, sorafenib, and pazopanib [81,82]	VEGF, VEGF receptor (VEGFR), fibroblast growth factor receptor 1, MMP2	Inhibit angiogenic pathways and reduce lesion size by activating apoptosis	Mice Rats
Quinagolide [83]	VEGF/VEGFR2 pathway	Shown to induce a considerable decrease in lesion size, potentially via regulation of angiogenesis	Humans

**Table 2:** List of pharmacological inhibitors targeting NF-κB and playing an important role in mitigating endometriosis.

Name of Inhibitor	Mainly Targeted Downstream/Upstream Proteins	Mode of Action	Study Model
Methyl ester of 2-cyano-3,12-dioxooleana-1,9-dien-28-oic acid [20]	NF-κB	Shows antioxidant and anti-inflammatory action as well as decreased angiogenesis and increased apoptosis in endometriotic lesions	Rats with surgically induced endometriosis
Dienogest [21]	NF-κB, TNF-α, IL-8	Attenuates expression of IL-8 by reducing TNFα-induced NF-κB activation and may confer a protective effect against endometriosis	Endometrial stromal cells (ESCs)
BAY 11-7085 and SN-50 [22]	NF-κB, adhesion molecule 1	Reduces ICAM-1 expression and cell proliferation and increases apoptosis of endometriotic lesions, thereby diminishing their development	Nude mice
Thalidomide [23]	NF-κB, TNF-α, IL-8	Attenuates the expression of IL-8 mRNA and protein by reducing TNF-α-induced NF-κB activation	Ectopic endometrial stromal cells (EESCs)
Genistein [24]	NF-κB, TNF-α, IL-6, IL-8	Inhibits expression of inflammatory mediators and decreases proliferation in mouse lesions	EESCs Mice
Ginsenoside [25]	NF-κB, protein kinase B	Suppresses endometriosis by reducing the viability of human ectopic endometrial stromal cells via the NF-κB signaling cascade	EESCs
Gossypol [26]	TNF-α, IL-1β	Induces regression of ectopic lesions via inhibition of estrogen receptor	Mice

**Table 4:** List of pharmacological inhibitors that target inflammatory molecules and play an important role in mitigating endometriosis.

Name of Inhibitor	Mainly Targeted Downstream/Upstream Proteins	Mode of Action	Study Model
Resveratrol [46]	IL-6, IL-1B, MCP-1	Downregulates expression of inflammatory markers in eutopic and, more markedly, ectopic endometrium	EESCs
Tocilizumab [35]	IL-6	Monoclonal anti-IL-6 antibody shown to lead to lesion regression in rats	Rats
Pyrvinium pamoate [47]	IL-6, IL-8	Suppresses mRNA expression of IL-6 and IL-8 in vitro	EESCs
Nobiletin [48]	NF-κB, IL-6, IL-1β	Reduces lesion size and pain by inhibiting cell proliferation, angiogenesis, and excess inflammation	Mice
(S,R)-3-(4-hydroxyphenyl)-4,5-dihydro-5-isoxazole acetic acid methyl ester [49]	MIF, IL-8, MCP-1	MIF inhibitor exhibits antiangiogenic effects in vitro	EESCs

**Table 7:** Pharmacological inhibitors targeting EMT.

Name of Inhibitor	Mainly Targeted Downstream/Upstream Proteins	Mode of Action	Study Models
Isoliquiritigenin [73]	E-cadherin, N-cadherin, Snail, Slug	Acts against viability, migration, and EMT in vitro. Reduces the volume and weight of mouse endometriotic lesions. Decreases the inflammatory response and triggers apoptosis	Endometrial cell lines Mice
Fucoidan [74]	Snail and Slug, Notch	Exerts anti-proliferative and anti-inflammatory effects, inhibiting EMT and inducing apoptosis	Endometrial cell lines Mice
Melatonin [69]	Notch homolog 1, Snail, Slug, N-cadherin, E-cadherin and Numb	Alleviates EMT and invasion by blocking estradiol and the Notch signaling pathway	Endometrial epithelial cells
3,6-dihydroxyflavone [75]	Notch signaling pathway	Reduces EMT and in vitro cell migration via inhibition of Notch and downstream molecules	EESCs

## Data Availability

Not applicable.
